# Experienced Adult Cochlear Implant Users Show Improved Speech Recognition When Target Fitting Parameters Are Applied

**DOI:** 10.1097/AUD.0000000000001513

**Published:** 2024-05-17

**Authors:** Richard de Quillettes, Marre Kaandorp, Paul Merkus, Sophia E. Kramer, Cas Smits

**Affiliations:** 1 Amsterdam UMC, Location Vrije Universiteit, Otolaryngology-Head and Neck Surgery, Ear and Hearing, Amsterdam Public Health research institute, Amsterdam, the Netherlands; 2 Amsterdam UMC, Location University of Amsterdam, Otolaryngology-Head and Neck Surgery, Ear and Hearing, Amsterdam Public Health research institute, Amsterdam, the Netherlands.

**Keywords:** Adults, Cochlear implants, Fitting, Speech recognition, Speech-in-noise

## Abstract

**Objectives:**

The aim of the present study was to investigate whether prediction models built by de Graaff et al. (2020) can be used to improve speech recognition in experienced adult postlingual implanted Cochlear CI users. de Graaff et al. (2020) found relationships between elevated aided thresholds and a not optimal electrical dynamic range (<50 CL or >60 CL), and poorer speech recognition in quiet and in noise. The primary hypothesis of the present study was that speech recognition improves both in quiet and in noise when the sound processor is refitted to match targets derived from the prediction models from de Graaff et al. (2020). A second hypothesis was that subjectively, most of the CI users would find the new setting too loud because of an increase in C levels, and therefore, prefer the old settings.

**Design:**

A within-participant repeated measures design with 18 adult Cochlear CI users was used. T- and C-levels were changed to “optimized settings,” as predicted by the model of de Graaff et al. (2020). Aided thresholds, speech recognition in quiet, and speech recognition in noise were measured with the old settings and after a 4-week acclimatization period with the optimized settings. Subjective benefit was measured using the Device Oriented Subjective Outcome Scale questionnaire.

**Results:**

The mean electrical dynamic range changed from 41.1 (SD = 6.6) CL to 48.6 (SD = 3.0) CL. No significant change in aided thresholds was measured. Speech recognition improved for 16 out of 18 participants and remained stable for 2 participants. Average speech recognition scores in quiet significantly improved by 4.9% (SD = 3.8%). No significant change for speech recognition in noise was found. A significant improvement in subjective benefit was found for one of the Device Oriented Subjective Outcome subscales (speech cues) between the old and optimized settings. All participants chose to keep the optimized settings at the end of the study.

**Conclusions:**

We were able to improve speech recognition in quiet by optimizing the electrical dynamic range of experienced adult CI users, according to the prediction models built by de Graaff et al. (2020). There was no significant change in aided thresholds nor in speech recognition in noise. The findings of the present study suggest that improved performance for speech recognition in quiet in adult Cochlear CI users can be achieved by setting the dynamic range as close as possible to values between 50 and 60 CL when the volume level is at 10.

## INTRODUCTION

Speech recognition abilities in quiet and in noise vary widely across adult cochlear implant (CI) users. Many factors contribute to this variation (Blamey et al. 1996; Lazard et al. 2012). Patient dependent factors such as age, duration of deafness, and etiology have been identified as significant parameters for variation in speech recognition (Lazard et al. 2012; Blamey et al. 2013). However, whereas many patient characteristics are fixed, other parameters may be modified or depend on specific decisions made by the clinicians. These adjustable parameters can be implantation related (e.g., brand and type of electrode), or related to rehabilitation methods and fitting parameters. Several studies have found that the positioning, insertion depth, and the number of inserted electrodes affect CI performance (Finley et al. 2008; Holden et al. 2013; O’Connell et al. 2016). Furthermore, auditory training can affect CI performance (Plant et al. 2015; Volter et al. 2020; Dornhoffer et al. 2022). Finally, fitting parameters are related to speech recognition performance (Loizou et al. 2000a, b; Busby & Arora 2016; Martins et al. 2021).

Validated prescriptive fitting rules (e.g., National Acoustic Laboratories, and Desired Sensation Level, fitting rules) are available as clinical guidelines for hearing aid (HA) fitting, however, such validated and generally accepted fitting rules are not available for CI fitting. The American Academy of Audiology cochlear implant clinical practice guideline provides recommendations on fitting adult CI users (Messersmith et al. 2019). Although the document emphasizes the importance of correct settings of threshold levels, upper levels and the electrical dynamic range, fitting rules (or targets) are not provided. It is stated that “Device programming is one of the most critical elements of a recipient’s success with a cochlear implant and is heavily influenced by the programming audiologist’s knowledge and experience with cochlear implants.” Given the absence of fitting rules, a large variation in fitting practices between CI centers and even within centers exists (Vaerenberg et al. 2014b; Browning et al. 2020; Wathour et al. 2021). There are some common practices: impedances are routinely measured every visit while some parameters, for example, sound coding strategy, stimulation mode, and loudness growth, are rarely modified (Vaerenberg et al. 2014b; Wathour et al. 2021; Maruthurkkara & Bennett 2022). The specific set of parameters stored in the sound processor is commonly called a MAP. In Cochlear CIs, normally, minimum (threshold, T-level) and maximum (comfort, C-level) current levels (CL) are set per electrode or electrode group. It is known that T- and C-levels, pulse rate, and pulse width influence speech recognition (Loizou et al. 2000b; Holden et al. 2011; Busby & Arora 2016; Martins et al. 2021). Currently, establishing threshold levels and upper stimulation levels are considered best practice when fitting CIs (Messersmith et al. 2019). Subjective patient feedback and psychophysical procedures are used where possible to determine these levels. But objective measurements as the electrically evoked compound action potential (ECAP) and the electrically evoked stapedial reflex threshold (ESRT), have also been investigated to use for determining these levels (Alvarez et al. 2010; Botros & Psarros 2010; Vargas et al. 2012; Holder et al. 2023). Some studies showed that the ECAP correlates to T- and C-levels (Thai-Van et al. 2004; Willeboer & Smoorenburg 2006; Botros & Psarros 2010). Therefore, by measuring the ECAP, profiles of these parameters across the electrodes could be determined. However, a systematic review of literature contradicted evidence for a correlation between the ECAP thresholds and behavioral T- and C-levels (de Vos et al. 2018). The ESRT is another objective measure used for fitting (Walkowiak et al. 2011; Çiprut & Adigül 2020). Several studies have shown strong correlations between ESRT and upper stimulation levels (Polak et al. 2006; Messersmith et al. 2019) and suggested that ESRTs are the most accurate approach to setting upper stimulation levels (Holder et al. 2023). Despite these results, a survey among CI audiologists throughout the United States showed that only 13% of the audiologists used ESRTs always or almost always for fitting adult CI users; 42% never used ESRTs (Browning et al. 2020).

Govaerts et al. (2010) used a different approach to fit CI sound processors. They used artificial intelligence in an outcome-driven approach to recommend and execute modifications to the fitting parameters. The program bases its recommendations on analyses of psychoacoustic test results along with current MAP settings. Meeuws et al. (2017) showed improvements in speech recognition at least 6 months after MAP modifications using this approach.

Most studies that have examined relationships between fitting parameters and outcomes have taken an experimental approach. Some others have retrospectively analyzed large clinical datasets and looked at associations between (fitting) parameters and outcomes (Van Der Beek et al. 2015; de Graaff et al. 2020; Caswell-Midwinter et al. 2022; Migliorini et al. 2024). Van Der Beek et al. (2015) analyzed data from a large set of Advanced Bionics CI users to determine population-based T- or M-level profiles to predict individual CI users’ T- and M-levels based on one psychophysical measurement. They reported significant correlations between speech recognition scores in quiet, and mean T-levels and electrical dynamic range. In a recent study with a large (N = 425) number of implanted ears with Advanced Bionics devices Caswell-Midwinter et al. (2022) reported that greater variation in basal impedance (electrodes 9 to 16) was associated with poorer word recognition in quiet. They also showed significant correlations between mean M-levels, variation of T- and M-levels, and word recognition in quiet. However, only the variation in basal impedance remained significant in the model accounting for other factors. de Graaff et al. (2020) conducted a study where the aim was to identify parameters that may improve fitting practices for Cochlear Nucleus sound processors. They built prediction models for speech recognition in quiet and in noise by using clinical data of 138 postlingually deaf adult Cochlear CI users. Separate analyses were performed for adult CI users with late and early onset of severe hearing impairment. The prediction models were built using fitting parameters and both objective- and psychoacoustic outcome measures. To provide clinicians with easy-to-use fitting guidelines, unchangeable factors such as age, etiology, and electrode position were not included in the models. A total of 33 parameters including aided sound field thresholds and parameters related to fitting (i.e., mean and variability of T- and C-levels, electrical dynamic range, ECAP thresholds, and impedances) were considered as independent variables. Several parameters were identified that predicted speech recognition in quiet and in noise. For the late onset CI users, models with adjustable parameters showed that mean electrical dynamic range (i.e., difference between T- and C-levels after compensation for volume settings) and mean aided thresholds were significant predictors of speech recognition in quiet. They took into account that for default settings, C-levels are reduced by 0 to 20% of the electrical dynamic range between the maximum (V = 10) and minimum volume settings (V = 1). For speech recognition in noise, only the mean aided threshold was identified as a significant adjustable parameter. They reported that the mean aided thresholds for octaves between 0.25 and 8 kHz should be below 24 dB SPL, and that mean electrical dynamic range over the entire electrode array should be between 50 and 60 CL when volume setting is at 10. Migliorini et al. (2024) also showed that larger mean electrical dynamic ranges were associated with better speech recognition scores in quiet, but in contrast with the results from de Graaff et al., they did not find an optimum value for the electrical dynamic range. While the prediction models from de Graaff et al. were able to predict speech recognition scores based on current settings, it was not investigated whether changing fitting parameters to these “optimal” settings, would in fact improve speech recognition in CI users. Furthermore, the ability of CI users to accept such “optimal” settings and acclimatize to them was not investigated. For many of the CI users, the change would result in a considerably louder sound processor setting. Because it is likely that some CI users may accept slightly louder settings but not the predicted optimal settings, we refer to these new settings as “optimized settings.”

The primary aim of the present study is to investigate whether the prediction models built by de Graaff et al. (2020) can be used to improve speech recognition in experienced adult postlingual implanted Cochlear CI users. Furthermore, the goal is to investigate whether acclimatization can help to accept “optimized” CI settings which, in general, will be louder than the old settings due to an increase in T-levels and/or increase in C-levels. To answer these questions, CI users with mean aided thresholds larger than 24 dB SPL, and a mean electrical dynamic range other than between 50 and 60 CL were refitted to match targets derived from the prediction models from de Graaff et al. Speech recognition scores in quiet and noise between old and “optimized” settings were compared. Subjective benefit was measured using a modified Device Oriented Subjective Outcome (DOSO) Scale questionnaire (Cox et al. 2014). The primary hypothesis was that speech recognition improves in quiet and in noise when fitting the sound processor with the predicted optimal settings. A second hypothesis was that subjectively, most of the CI users find the new setting unpleasantly loud, and therefore, prefer a softer setting, even though speech recognition is poorer.

## MATERIALS AND METHODS

### Participants

Eighteen adult unilateral CI users (12 males, 6 females) were included in this study. Table 1 lists the characteristics of the study population. Inclusion criteria were equal to those of the late onset (LO) group used by de Graaff et al. (2020) because the fitting targets for the present study were based on the prediction model from their study. All participants were postlingually deafened adult CI users with late onset (i.e., after the age of 7) of severe hearing impairment. Furthermore, they all had at least 1 year of experience with their CI and had no additional relevant medical problems that could interfere with testing (e.g., severe cognitive impairment or physical problems that made traveling to the clinic difficult). Participants were using a Cochlear sound processor from the CP900 series (n = 3), or CP1000 series (n = 15) with full insertion and 22 active electrodes. Only CI users with standard map parameters, that is, advanced combination encoder speech coding strategy, 8 maxima, 900 Hz pulse rate, 25 µsec pulse width, 25 dB T-SPL, and 65 dB C-SPL, were included. Participants were included if they had MAP parameters to be less than optimal, as predicted by the model of de Graaff et al.: aided thresholds of at least 27 dB HL, averaged over octave frequencies from 250 Hz to 8 kHz (13 out of 18 participants), and/or a mean electrical dynamic range of less than 50 CL (16 out of 18 participants) or more than 60 CL (0 out of 18 participants) (Table 1). Note that the model of de Graaff et al. predicted maximal speech recognition for mean aided thresholds <24 dB HL, but the difference between <24 dB HL and 24 to 27 dB HL was not significant.

**TABLE 1.** Demographic variables and mean aided thresholds and electrical dynamic range with old and “optimized” settings

Participant	Sex	Age (yr)	Old Settings		“Optimized” Settings		Difference (Optimized—Old)	
			Mean Threshold (dB HL)	Mean DR (CL)	Mean Threshold (dB HL)	Mean DR (CL)	T Levels (CL)	Mean DR (CL)
1	M	78	30	39	30	46	6	7
2	M	77	24	36	25	50	0	14
3	M	79	23	35	28	40	0	5
4	F	79	32	44	29	45	9	1
5	M	53	25	36	30	51	1	15
6	M	35	27	35	27	50	2	15
7	M	60	29	46	28	50	6	4
8	F	66	29	41	32	50	5	9
9	M	79	28	53	33	50	6	−3
10	M	78	28	31	30	43	3	12
11	F	54	28	40	25	50	4	10
12	M	82	31	53	29	50	9	−3
13	F	60	25	49	25	50	1	1
14	F	64	27	45	29	50	3	5
15	M	69	26	37	23	50	2	13
16	M	73	27	33	29	50	2	17
17	F	75	34	44	33	50	11	6
18	M	70	28	43	24	50	4	7

The study was approved by the Medical Ethical Committee of VU University Medical Center, Amsterdam (20.016). All participants gave written informed consent.

### Speech Recognition Tests

For measuring speech recognition in quiet, Dutch consonant-vowel-consonant (CVC) words were used. In each test condition four lists consisting of 12 meaningful monosyllables, uttered by a female speaker, were used (Bosman & Smoorenburg 1995). The words were presented through a single loudspeaker (Yamaha MSP5 Studio) at a distance of approximately 70 cm in front of the participants. The lists were randomized per participant, but to avoid the effects of inter-list variability (de Graaff et al. 2018; Polspoel et al. 2024), for each participant the same lists from the first session were used in the second session (after 4 weeks) to compare scores between different sound processor settings. Note that the participants were all very familiar with these speech recognition test material because it is the standard test in the Netherlands for speech audiometry, thus no learning effects were expected.

Speech recognition in noise was measured using the digits-in-noise test (DIN) (Smits et al. 2013; Kaandorp et al. 2015; de Graaff et al. 2018). This test presents lists of 24 broadband, homogeneous digit-triplets in long-term average speech spectrum masking noise. The test measures the speech recognition threshold (SRT), the signal to noise ratio (SNR) at which 50% of the digit triplets are correctly recognized, via a one-up, one-down adaptive procedure. Stimuli were presented via the same loudspeaker as used for the CVC words, at an overall level of 65 dB SPL. Three lists were measured per test condition.

### Subjective Benefit

Subjective benefit was assessed using a translated and modified version of the Device Oriented Subjective Outcome Scale (DOSO) questionnaire (Cox et al. 2014). This English validated questionnaire consists of 40 questions on a Likert scale, normally used to measure the subjective benefit of HAs. Scoring is as follows. For the three items in the “Use” subscale, numbers from 1 to 5 are assigned to the five alternatives. For items with the 7-point response scale, numbers are assigned to the responses (not at all = 1; a little = 2; somewhat = 3; medium = 4; considerably = 5; greatly = 6; and tremendously = 7). For all items, a higher number is associated with a better outcome. A score is computed for each of the six subscales (speech cues, listening effort, pleasantness, quietness, convenience, and use) by averaging the scores for the items within that subscale (Cox et al. 2014). An example question of the original DOSO is: “How good are the hearing aids at....? Keeping loud sounds from being uncomfortable?.” The original questionnaire was translated to Dutch using the Forward-Backward-Translation method (Beaton et al. 2000). Question one was omitted because it referred to the whistling of HAs and the questionnaire was modified to fit CI use (see Date file for the modified Dutch DOSO questionnaire in Supplemental Digital Content, http://links.lww.com/EANDH/B397).

### MAP Settings

T- and C-levels were changed to “optimal settings,” as predicted by the model by de Graaff et al. (2020). It means that the target was to achieve mean aided thresholds of less than 24 dB HL and a mean electrical dynamic range between 50 and 60 CL for a volume level set at 10. We decided to apply global changes to map parameters to try to achieve this, because this is the easiest to implement in clinical practice and we expected smaller effects on the sound quality than with adjustments of individual electrodes. First, mean-aided thresholds with the current settings were compared with the target thresholds. Then T-level adjustments were estimated using


$$\begin{array}{l} \begin{array}{l} T_{new}=T_{current}+\left(\frac{DR_{current}}{40}\times \left(FF_{current}-24\right)\right) \end{array} \end{array}$$ (1)

In which $FF_{current}$ is the mean of the current aided thresholds, $DR_{current}$ is the mean of the current dynamic range, $T_{current}$ is the mean of the current T-levels, and $T_{new}$ is the mean of the new T-levels.

Equation (1) was derived from the approximately linear relationship between the sound processor output, expressed in CLs, and the input, expressed in dB SPL. For default T-SPL (25 dB) and C-SPL (65 dB) levels a 1-dB change in input level yields an average change of DR_current_/40 in CLs. The true relationship is nonlinear and depends on the *Q*-factor (Vaerenberg et al. 2014a), but the approximation is reasonable for standard values of the *Q*-factor (~20) and ensures practical applicability. After setting the new T-levels, C-levels were chosen such that the mean electrical dynamic range was between 50 and 60 CL. It means that if the new dynamic range was lower than 50 CL, C-levels were raised until the dynamic range was 50 CL. If the DR was above 60 CL, C-levels were lowered until dynamic range was 60 CL. In case the new settings were too loud initially, progressive maps with increasing dynamic range (e.g., dynamic ranges of 46, 48, and 50 CL, respectively) were created by varying C-levels. Note that the electrical dynamic range was reduced to 50 CL for 2 participants because the electrical dynamic range was larger than 50 CL with the old settings, but less than 50 CL after increasing T-levels. Progression of the maps was based on the new dynamic range, where each progressive map increased the dynamic range by one third of the total increase.

### General Procedure

A within-participant repeated measures design was used. Participants visited the clinic twice with each visit taking approximately 90 minutes. During the first visit CI microphone covers were replaced. Speech recognition in quiet (four lists), in noise (three lists), and aided thresholds (narrow band noise) were then measured with the original CI settings. The participants were accustomed to these settings and the results were used in the analyses, which are referred to as measurements with “old settings.” Participants did not wear the contralateral HA during speech recognition testing when fitted bimodal. They also filled in the DOSO questionnaire. Next, the new (“optimal”) MAP settings were programmed in their sound processor. Volume control was disabled when applicable. They were told that the “optimal” settings might be loud and were counseled to try them because of the expected improvement in speech recognition. In case new settings were not acceptable for them (i.e., too loud; 7 out of 18 participants), participants were given three progressive MAPs in three program slots with the optimal setting in the third program slot. These participants were able to switch between programs and were instructed to switch to the following program after 7 days or as quickly as possible. Finally, during the first visit, speech recognition was tested with the new settings (optimized settings) which was not the optimal setting for all participants due to the reason explained earlier. Once again aided thresholds, four CVC lists, and three DIN lists were measured.

There was a 4-week interval between both visits during which participants used the new MAP settings. They used the contralateral HA during this period if bimodal fitted. If participants were unable to accept the new settings immediately and thus were given progressive MAPs, they were contacted after 2 weeks. Their progress was discussed and they were counseled to work toward the optimal settings.

Speech recognition and aided thresholds were measured after the 4-week acclimatization period with the new settings during the second visit, which are referred to as measurements with “optimized settings.” Again participants filled out the DOSO questionnaire. Old settings were then restored and aided thresholds and speech recognition were measured once more. Daily device use was collected from data logging at the first and second visits. Finally, participants were asked which settings, old or optimized, they preferred.

### Analysis

The data were analyzed using IBM SPSS (version 28). CVC scores, SRT’s, and aided thresholds measured at the first visit with old settings and measured at the second visit with optimized settings, after 4 weeks of acclimatization, were used for analyses. Speech in quiet scores were transferred from percentage scores to rationalized arcsine units (RAU) to normalize variance (Studebaker 1985) before statistical testing for the difference in mean scores. No transformation was applied to SRT values for speech in noise scores. SRTs and CVC scores were averaged per measurement condition per participant and these average scores were used in the analyses. The DOSO data were divided into six subscales (Cox et al. 2014) for analysis. There were instances of missing data in certain subscales of the DOSO questionnaire. Due to the absence of a defined protocol for handling missing data in the DOSO, we adopted the following approach: If more than one item was missing in subscales comprising three, four, or five items, the participant’s data for that subscale (in both DOSO questionnaires) were excluded. No data were excluded for the speech cues subscale, as each participant completed more than 9 out of 14 items. For the listening effort subscale, three data points were excluded due to only one item being completed; the remaining data points were derived from a minimum of 9 out of 10 items. The Wilcoxon signed-rank test was used to test the significance of the differences between the first and second scores on each DOSO subscale. The significance of the differences between the average CVC scores, SRT’s, and aided thresholds measured at the first visit with old settings and the second visit with optimized settings were tested with paired *t* tests. Normal *Q*-*Q* plots were visually inspected to verify a normal distribution of the data and Kolmogorov–Smirnov tests were used to further confirm a normal distribution. The relationship between the change in CVC score and the change in SRT was assessed using Pearson correlation coefficient. In addition the differences between the CVC scores between old settings and optimized settings for each participant were tested. To account for the different recognition probabilities of phonemes in a list, an advanced version of the approach from Thornton and Raffin (1978) was used (Holube et al. 2020; Polspoel et al. 2024). Differences between SRTs with old settings and optimized settings were also tested for each participant using an estimated standard error of measurement of 1.1 dB (Kaandorp et al. 2015).

## RESULTS

The mean aided threshold averaged across all participants and frequencies was 27.8 (SD = 2.8, range = 23 to 34) dB HL with old settings. Using Eq. (1) separately for each participant yielded a small increase in mean T-levels from 118 (SD = 18) CL for the old settings to 122 (SD = 18) CL for the optimized settings. The mean aided threshold averaged across all participants for the optimized settings was 28.3 (SD = 3.0, range = 23 to 33) dB HL where an average threshold of 24 dB HL was expected. There was no significant difference in aided thresholds between old settings and optimized settings, *t*(17) = −0.65, *p* = 0.523. A change in dynamic range to at least 50 CL was achieved in 14 out of 16 participants who started with a dynamic range below 50 CL. None of the participants started with a dynamic range larger than 60 CL. The mean dynamic range over all participants increased significantly from 41.1 (SD = 6.5) CL to 48.6 (SD = 3.0, range = 40 to 51) CL, *t*(17) = −5.16, *p* < 0.001.

**Fig. 1.**
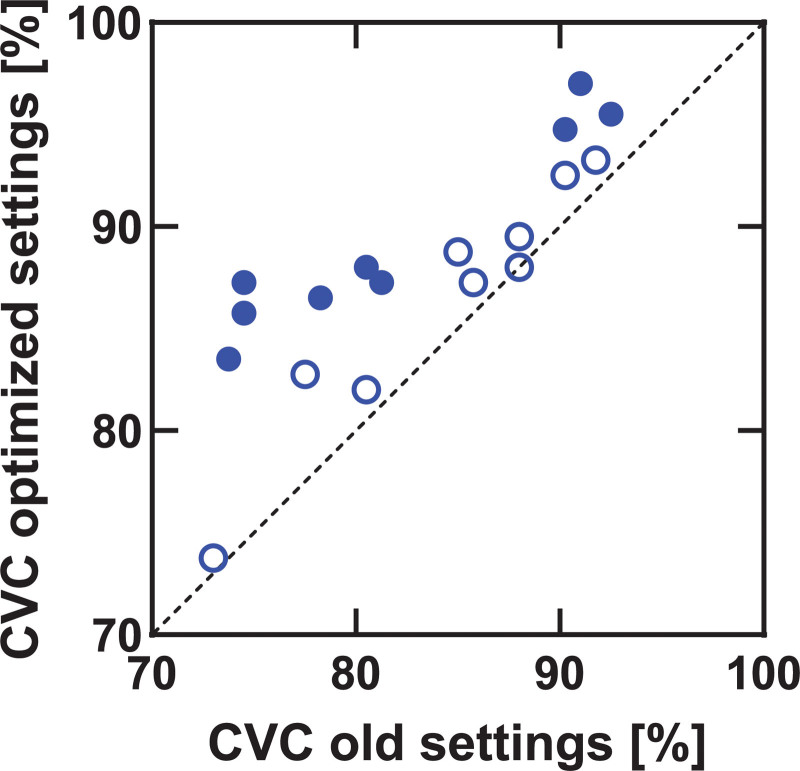
Speech recognition in quiet scores with optimized settings against scores with old settings. All scores above the dotted line indicate an improvement with optimized settings. Filled symbols represent significant differences between optimized and old settings for individual participants. CVC indicates consonant-vowel-consonant.

**Fig. 2.**
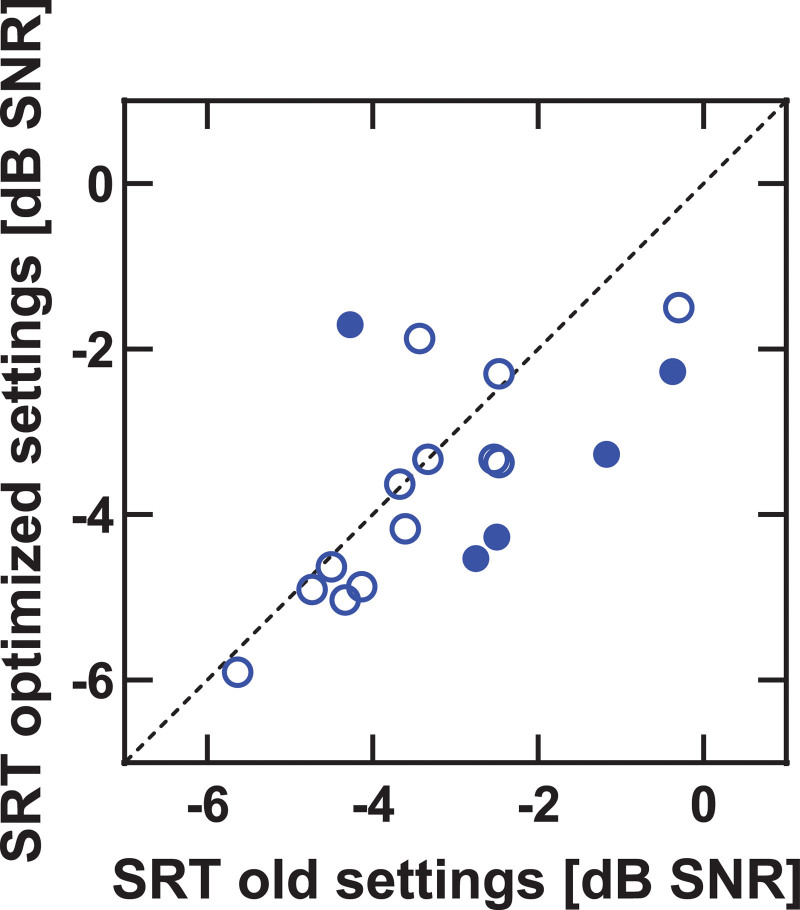
Speech recognition in noise scores with optimized settings against scores with old settings (SRT). All scores below the dotted line indicate an improvement with new settings (lower SRTs indicate better performance). Filled symbols represent significant differences between optimized and old settings for individual participants. SRT indicates speech recognition threshold.

**Fig. 3.**
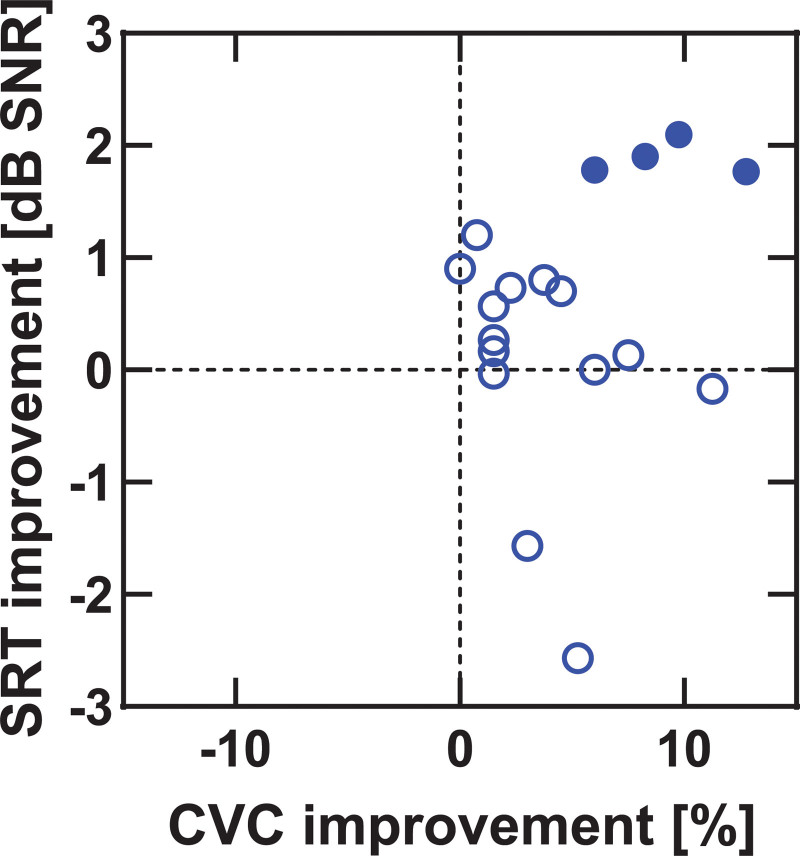
Improvements in speech recognition in noise vs. improvement in speech recognition in quiet between old and optimized settings. Filled symbols represent significant differences between optimized and old settings for individual participants. SRT indicates speech recognition threshold.

Figure 1 shows the effect of MAP changes on speech recognition scores in quiet. An improvement in CVC score was found for 16/18 participants. Filled symbols in Figure 1 represent significant improvements for individual participants. The mean improvement over all participants was 4.9% (SD = 3.8%, range = 0 to 12.8), from 83.1 to 88.0%. The difference between the mean CVC scores, transformed into RAU values, with old settings and with optimized settings was statistically significant, *t*(17) = −6.01, *p* < 0.001. The results for speech recognition in noise are shown in Figure 2. Again, filled symbols, represent significant changes for individual participants. The mean SRTs were −3.1 (old settings) and −3.6 dB SNR (optimized settings) with no statistically significant difference between both, *t*(17) = 1.7, *p* = 0.10. Note that lower SRTs indicate better performance. Figure 3 shows the improvement in speech recognition in noise (SRT) against the improvement in speech recognition in quiet (CVC score). The correlation between both measures (ρ=−0.18) was not statistically significant (*p* = 0.47).

**Fig. 4.**
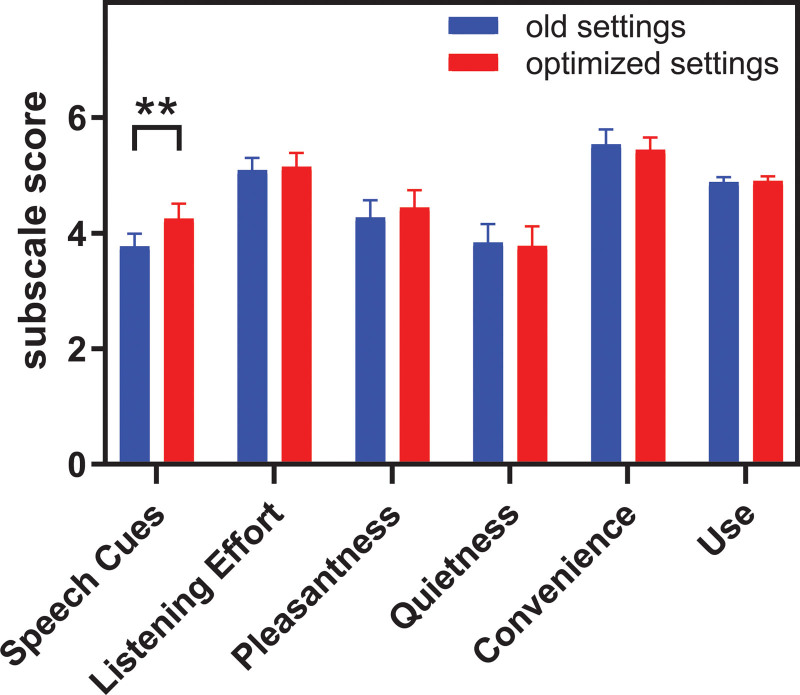
Subjective benefit comparison between old and optimized settings for each of the subscales of the DOSO questionnaire. A higher score indicates a better outcome. The maximum score for the first five categories is 7; the maximum score for category “Use” is 5. DOSO indicates Device Oriented Subjective Outcome. ** *p* < 0.01.

The results of the subjective benefit measured using the DOSO questionnaire are shown in Figure 4. A Wilcoxon signed-rank test indicated that the mean score on the DOSO subscale speech cues was significantly higher with optimized settings than with the old settings, *z* = −2.82, *p* = 0.005. No significant difference in scores was found for the other subscales.

**Fig. 5.**
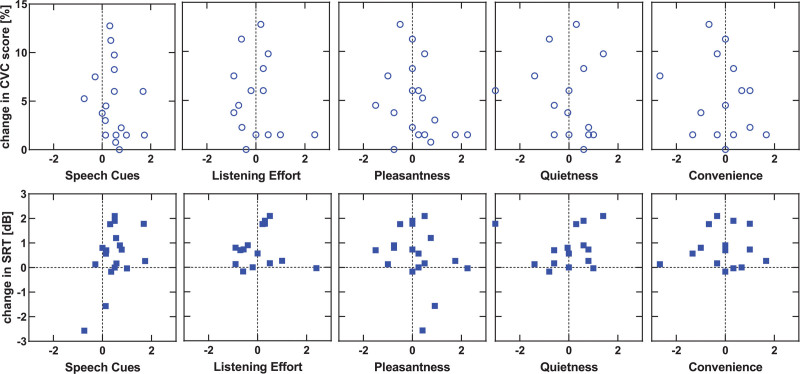
Change in speech recognition in quiet (CVC) scores (upper row) and change in speech recognition in noise (lower row) against change in DOSO subscale score. Positive numbers mean improvements. CVC indicates consonant-vowel-consonant; DOSO Device Oriented Subjective Outcome.

To further explore the relationship between subjective (i.e., DOSO scores) and objective (i.e., speech recognition in quiet and noise) measures for individual participants, Figure 5 shows the change in DOSO subscale scores against the change in CVC recognition (upper row) and against the change in SRT (lower row). It suggests that the change in sound processor settings affects the ability to detect and understand speech in specific situations, represented by the speech cues subscale, for most of the participants (16/18 participants). For the other subscales, changes are approximately evenly distributed around zero. The mean daily device use did not differ between the first visit (*M* = 14.3 hours) and second visit (*M* = 14.6 hours), *t*(17) = −1.88, *p* = 0.078. The correlation between daily device use (hour per day) at first visit (old settings) and second visit (optimized settings) was strong (ρ=0.985, *p* < 0.001).

Eventually, all participants chose to keep the optimized settings at the end of the second visit.

## DISCUSSION

The aim of the present study was to investigate whether the prediction models built by de Graaff et al. (2020) can be used to improve speech recognition in quiet and in noise in adult postlingual implanted experienced Cochlear CI users. Furthermore, our aim was to investigate whether CI users choose and accept these “optimized” CI settings or choose more comfortable (i.e., softer) settings at the expense of lower speech recognition in quiet and noise. The results of the study by de Graaff et al. suggested that target values for the mean aided thresholds and mean electrical dynamic range exist. We have fit participants to these target values by adjusting global T-levels and global C-levels and found a statistically significant improvement for speech recognition quiet, whereas no significant improvement was found for speech recognition in noise. A DOSO questionnaire revealed a statistically significant change in one of the subscales (speech cues).

The mean electrical dynamic range changed significantly from 41.1 to 48.6 CL, while no significant change in aided thresholds was measured. The results from speech recognition testing with old and optimized settings confirmed our first hypothesis: average speech recognition scores in quiet improved significantly by 4.9% (SD = 3.8%) for CVC words. We did not find a deterioration in speech recognition in quiet for any of the participants. For speech recognition in noise, with mean SRTs changing from −3.1 to −3.6 dB SNR, no significant improvement was found between scores with old and optimized settings. This, however, is not at odds with model predictions because we did not find a significant improvement in aided thresholds. Figure 2 suggests that 2 participants may perform worse in noise with the optimized settings. Inspection of the individual test results revealed that both participants had very poor results on the first of the three DIN tests with the optimized settings. We could not find a reason for this observation other than random error, but these possible outliers could explain the result. The measured amount of improvement for our study participants is in line with the predicted improvement from the models built by de Graaff et al. The model predicted that when changing the dynamic range from <40 CL to 50 to 60 CL (i.e., an increase of at least 10 CL in dynamic range), speech recognition would improve by 10.2 RAU. In the present study, the average dynamic range changed by approximately 8 CL (from 41 to 49 CL) resulting in an average speech recognition improvement of 6.5 RAU (4.9%). Ideally, the minimum (and mean) dynamic range in this study would have been over 50 CL, but not all participants were able to acclimatize to this loudness. Sixteen out of 18 participants started with a mean dynamic range of less than 50 CL, and 14 were able to acclimatize to settings with a dynamic range of 50 CL. The 4 participants who were unable to make it to the optimal settings ended on the second program, increasing the dynamic range with 67% of the difference between both settings. It could be that not all CI users accept optimal settings due to unpleasant loudness of the CI, due to loudness summation with bimodal stimulation, or because they need a longer acclimatization period. One of the participants used the CI less than 12 hours per day (on average 6 hours per day) which may explain the difficulty in acclimatizing to the optimized settings. Studies have shown a positive correlation between hours of processor use per day and speech recognition scores (Holder et al. 2020; Holder & Gifford 2021). Because the daily device use is relatively long for the participants in our study (*M* = 14.6 hours per day), we do not expect that this is an important factor in our study. The model by de Graaff et al. suggested that both aided thresholds and the dynamic range are important factors for speech recognition. For most of the adult CI users in our clinic, it means that the largest benefit is expected from increasing the dynamic range by increasing C-levels. An even further increase in C-levels is needed when T-levels have to be increased to improve aided thresholds. The effect of a change in the electrical dynamic range on speech recognition has been studied before, but mainly by changing T-levels (Busby & Arora 2016; Rader et al. 2018; Martins et al. 2021) or lowering C-levels (Martins et al. 2021). The main reason why less research has been done on the effect of increasing C-levels on speech recognition is that, when properly fitted, C-levels correspond to comfortable levels. It is expected that further increasing C-levels results in uncomfortable loud sound perception. The results from the present study however suggest that standard procedures to determine C-levels (see de Graaff et al. 2020 for a detailed description of the standard fitting procedures in our clinic) often result in nonoptimal settings due to too small dynamic ranges. This suggestion is consistent with a study from Holder et al. (2023) who reported higher upper stimulation levels and higher speech recognition scores for maps based on ESRTs than for maps based on behavioral thresholds. Pitt et al. (2021) also reported systematically higher ESRTs than behavioral upper thresholds, but a relationship with speech recognition scores was not presented. It would be an interesting research topic to see whether C-levels corresponding to an electrical dynamic range of 50 CL correspond to C-levels based on ESRT measurements.

We did not find a significant effect of changes in T-levels on aided thresholds. This finding is not unexpected given the relatively small mean increase in T-levels of 4 CL. Busby and Arora (2016) reported a mean decrease in aided thresholds of 1.7 dB and an increase of 4.1 dB when applying respectively 30% compression and expansion to the electrical dynamic range. The 30% compression and expansion corresponds to a mean change in T-levels of 11.7 CL (i.e., 30% of the mean electrical dynamic range of 39 CL in their study population). Martins et al. (2021) showed that increasing or decreasing T-levels by 10 CL, decreased and increased mean aided threshold with 1.7 and 1.2 dB, respectively. Several factors can explain why we did not find the expected change in aided thresholds. First, the exact relationship between aided thresholds, T-levels, dynamic range, *Q*-factor, T-SPL, etc. is not known. The assumed linear relationship between CL and input level is an appropriate approximation but the largest deviations occur near the end of the range, which are exactly the levels that we manipulated. For the default value *Q* = 20, the relationship between sound processor output and input is slightly curved (concave down, Vaerenberg et al. 2014a), thus a greater change in T-level is probably needed to achieve the target aided thresholds. Second, the measurement error for pure-tone thresholds is relatively large compared with the predicted shift (Leijon 1992). Third, the so-called T-tail, a limited or slow loudness growth over a portion of the lower part of the dynamic range (Wolfe 2018). If T-levels of some of the participants were in a T-tail, then a small change in T-level has no or little effect on aided threshold. A possible solution would have been to determine optimal thresholds iteratively, by changing T-levels, then determining thresholds, changing T-levels again, and so on. We judged such a method as too complicated for clinical use and too effortful for our participants. More advanced procedures to determine T-levels could be helpful (Rader et al. 2018). The drawback of increasing T-levels to lower aided thresholds is that it decreases the dynamic range which negatively affects speech recognition. Thus, this complex relationship must be taken into account.

The results did not confirm our second hypothesis. We had expected that most of the CI users would find the optimized setting unpleasantly loud, and would prefer a softer setting, even though speech recognition would be poorer. The DOSO questionnaire showed a significant difference for the subscale speech cues between the old and optimized settings which is in good agreement with the change in speech recognition. Despite many users reporting that the optimized settings were very loud initially, no significant difference on the DOSO subscale pleasantness was found. This may be because participants got acclimatized to new louder settings after a 4-week period. Seven participants needed multiple MAPs to acclimatize but 11 participants immediately accepted the optimized settings. Many participants reported a decrease in listening effort with the optimized settings which is not reflected in a change in the DOSO subscale listening effort. The reported improvement is likely related to the improvement in speech recognition (Zekveld et al. 2010). One participant even reported that he/she no longer needed to look at faces to understand speech. Contrary to our hypothesis, all participants chose to keep the optimized settings at the end of the study.

The present study shows that adjusting global fitting parameters to “target,” that is, changing T-levels based on aided thresholds and Eq. (1), and C-levels to achieve an electrical dynamic range of 50 CL, improves speech recognition in experienced adult CI users. It suggests that raising the C-levels and thereby increasing the electrical dynamic range improves speech recognition even when the target value of 50 to 60 CL cannot be reached. This is in line with the model predictions from de Graaff et al. (2020) that suggested an increase in speech recognition with an increase in electrical dynamic range. A further increase above 60 CL yields a decrease in speech recognition according to the model. A limitation in the present study was the relatively small number of participants and applying inclusion criteria. We only included postlingually deafened adult CI users with late onset of severe hearing impairment. The results may therefore be different for CI users with early onset of hearing impairment (de Graaff et al. 2020). Because only those CI users were eligible for which relatively large improvements were expected, and because of the limited pool of eligible adult Cochlear CI users, recruiting a significantly higher number of participants was not feasible. Despite the small number of participants, the results across the study were consistent. A second limitation is that we don’t know whether our results generalize to populations from other CI centers. We expect that our results are robust for speech recognition in quiet and in noise, but future research in other CI centers or a multicenter study should confirm this.

### Clinical Relevance

The findings of the present study suggest that improvements in speech recognition in quiet in adult Cochlear CI users can be achieved by setting the electrical dynamic range to values between 50 and 60 CL when the volume level is at 10. For the volume level at 6, these values correspond to approximately 55 and 66 CL, respectively. Having such a target for CI fitting can reduce variation across CI centers and can lead to more uniform fitting practices. We recommend that when increasing the electrical dynamic range to at least 50 CL in the clinic, an appropriate acclimatization period and loudness build up is given. The time frame is dependent on the needed change, but should be at least 4 weeks.

## CONCLUSION

In conclusion, we were able to improve speech recognition in quiet by optimizing the electrical dynamic range of experienced adult CI users, according to the prediction models built by de Graaff et al. (2020). Despite changes in T-levels, there was no significant change in aided thresholds and speech recognition in noise. Participants were able to acclimatize to new settings and no subjective differences between settings after a 4-week period were found, despite large differences in initial subjective loudness. All patients kept on using their new setting after the study. More research is needed to further investigate the effects of changing aided thresholds on speech recognition in quiet and in noise, and the relationship between the optimal electrical dynamic range and ESRTs.

## ACKNOWLEDGMENTS

The study was approved by the Medical Ethical Committee of VU University Medical Center, Amsterdam (20.016, PI Cas Smits).

## Supplementary Material
