# Supplementary material for: Experienced Adult Cochlear Implant Users Show Improved Speech Recognition When Target Fitting Parameters Are Applied
Source: Ear Hear. 2024 May 17;45(5):1264–73. doi: 10.1097/AUD.0000000000001513 (PMC11325977; doi:10.1097/AUD.0000000000001513)
Supplement: Supplementary file 1 [file aud-45-1264-s001.pdf]

DOSO (modified for CI)

Name: ______________________________ Date of Birth: ___________ Today's Date: ___________

This questionnaire measures how well your CI works. Please read each question and circle one letter to show the answer that is closest to your opinion.

The guide shown on the right describes the meaning of each letter.

A Not at all
B A little
C Somewhat
D Medium
E Considerably
F Greatly
G Tremendously

How good is your CI at…

1	Providing a pleasing sound quality?	A	B	C	D	E	F	G
2	Making loud speech clear?	A	B	C	D	E	F	G
3	Making music pleasant?	A	B	C	D	E	F	G
4	Eliminating the need to have someone else explain what was said?	A	B	C	D	E	F	G
5	Making other people's voices sound clear in a moving car?	A	B	C	D	E	F	G
6	Making children's voices understandable?	A	B	C	D	E	F	G
7	Making your voice sound natural to you?	A	B	C	D	E	F	G
8	Catching the beginning of sentences?	A	B	C	D	E	F	G
9	Picking up overhead announcements in stores?	A	B	C	D	E	F	G
10	Catching your name being called in a waiting room?	A	B	C	D	E	F	G
11	Making the batteries easy to change?	A	B	C	D	E	F	G
12	Picking up speech when the talker's lips are not visible?	A	B	C	D	E	F	G
13	Keeping background noise to a minimum?	A	B	C	D	E	F	G
14	Catching what waiters say in a busy restaurant?	A	B	C	D	E	F	G
15	Catching what someone says on the first try?	A	B	C	D	E	F	G
16	Cutting out background noise in a restaurant?	A	B	C	D	E	F	G

17	Picking up soft sounds that follow loud ones?	A	B	C	D	E	F	G
18	Making speech clear in a face-to-face conversation?	A	B	C	D	E	F	G
19	Not using up batteries too fast?	A	B	C	D	E	F	G
20	Picking up what strangers say the first time?	A	B	C	D	E	F	G
21	Keeping the sound of your voice comfortable to you?	A	B	C	D	E	F	G
22	Improving enjoyment of everyday activities?	A	B	C	D	E	F	G
23	Catching the words when someone speaks from another room?	A	B	C	D	E	F	G
24	Picking up what someone says across a large room?	A	B	C	D	E	F	G
25	Being easy to put in and take out of your ears?	A	B	C	D	E	F	G
26	Picking up sounds that are missed without them?	A	B	C	D	E	F	G
27	Making loud music tolerable?	A	B	C	D	E	F	G
28	Catching a person's name when they are introduced?	A	B	C	D	E	F	G
29	Recognizing different voices?	A	B	C	D	E	F	G
30	Reducing misunderstandings during conversations?	A	B	C	D	E	F	G
31	Making the television sound clear?	A	B	C	D	E	F	G
32	Making conversation easier?	A	B	C	D	E	F	G
33	Keeping wind noise from being annoying?	A	B	C	D	E	F	G
34	Keeping the volume at a pleasing level?	A	B	C	D	E	F	G
35	Distinguishing between male and female voices?	A	B	C	D	E	F	G
36	Keeping loud sounds from being uncomfortable?	A	B	C	D	E	F	G

37 How many days a week do you *usually* wear your CI?

- ☐ None
- ☐ 1-2 days
- ☐ 3-4 days
- ☐ 5-6 days
- ☐ Every day

38 On the days you use your CI, how many hours do you usually wear it?

- ☐ 1-4 hrs.
- ☐ 5-6 hrs.
- ☐ 7-8 hrs.
- ☐ 9-10 hrs.
- ☐ 11 hrs. or more

39 In situations where you need to improve your hearing, how often do you wear your CI?

- ☐ Always (100%)
- ☐ Usually (75%)
- ☐ Sometimes (50%)
- ☐ Rarely (25%)
- ☐ Never (0%)

DOSO vragenlijst

Proefpersooncode:
Datum van vandaag:

Deze vragenlijst meet hoe goed uw CI werkt. Lees elke vraag en omcirkel de letter die het beste overeenkomt met uw mening

Hiernaast staat de betekenis van elke letter.

A. Helemaal niet
B. Een beetje
C. Enigszins
D. Gemiddeld
E. Behoorlijk
F. Zeer
G. Enorm

Hoe goed is uw CI in het…

1.	leveren van een aangename geluidskwaliteit?	A B C D E F G
2.	duidelijk maken van luide spraak?	A B C D E F G
3.	aangenaam maken van muziek?	A B C D E F G
4.	overbodig maken dat iemand anders moet uitleggen wat er gezegd is?	A B C D E F G
5.	duidelijk laten klinken van stemmen van anderen in een rijdende auto?	A B C D E F G
6.	verstaanbaar maken van kinderstemmen?	A B C D E F G
7.	zorgen dat uw stem natuurlijk voor u klinkt?	A B C D E F G
8.	opvangen van het begin van zinnen?	A B C D E F G
9.	opvangen van berichten die omgeroepen worden in een winkel?	A B C D E F G
10.	horen van uw naam als u geroepen wordt in een wachtkamer?	A B C D E F G
11.	makkelijk kunnen vervangen van (oplaadbare) batterijen?	A B C D E F G
12.	opvangen van spraak als de lippen van de spreker niet zichtbaar zijn?	A B C D E F G
13.	het zo veel mogelijk beperken van het achtergrondgeluid?	A B C D E F G
14.	opvangen van wat obers zeggen in een druk restaurant?	A B C D E F G
15.	opvangen wat iemand zegt bij de eerste poging?	A B C D E F G
16.	wegfilteren van achtergrondruis in een restaurant?	A B C D E F G

17.	opvangen van zachte geluiden die volgen na harde geluiden?	A B C D E F G
18.	duidelijk maken van spraak in een één-op-één gesprek?	A B C D E F G
19.	niet te snel opmaken van batterijen?	A B C D E F G
20.	opvangen van wat vreemden zeggen de eerste keer?	A B C D E F G
21.	comfortabel houden van uw eigen stem voor uzelf?	A B C D E F G
22.	plezier in alledaagse activiteiten verbeteren?	A B C D E F G
23.	opvangen van de woorden als iemand spreekt vanuit een andere kamer?	A B C D E F G
24.	opvangen van wat iemand zegt aan de andere kant van een grote kamer?	A B C D E F G
25.	makkelijk maken van het op en af doen?	A B C D E F G
26.	opvangen van geluiden die u zonder CI zou missen?	A B C D E F G
27.	tolereerbaar maken van harde muziek?	A B C D E F G
28.	opvangen van iemands naam wanneer een persoon aan u wordt voorgesteld?	A B C D E F G
29.	herkennen van verschillende stemmen?	A B C D E F G
30.	verminderen van misverstanden tijdens gesprekken?	A B C D E F G
31.	duidelijk laten klinken van de televisie?	A B C D E F G
32.	gemakkelijker maken om gesprekken te voeren?	A B C D E F G
33.	zorgen dat windruis niet vervelend is?	A B C D E F G
34.	het volume op een aangenaam niveau houden?	A B C D E F G
35.	onderscheid maken tussen mannelijke en vrouwelijke stemmen?	A B C D E F G
36.	zorgen dat harde geluiden niet oncomfortabel worden?	A B C D E F G
37.	hoeveel dagen per week draagt u uw CI gewoonlijk?	☐ Geen<br>☐ 1-2 dagen<br>☐ 3-4 dagen<br>☐ 5-6 dagen<br>☐ Elke dag

38. op de dagen dat u uw CI gebruikt, hoeveel uur per dag draagt u het dan gewoonlijk?

- ☐ 1-4 uur
- ☐ 5-6 uur
- ☐ 7-8 uur
- ☐ 9-10 uur
- ☐ 11 uur of meer

39. in situaties waar u beter moet horen, hoe vaak draagt u dan uw CI?

- ☐ Altijd (100%)
- ☐ Meestal (75%)
- ☐ Soms (50%)
- ☐ Zelden (25%)
- ☐ Nooit (0%)
